# Evaluation of electroacupuncture acupoint selection’s potential in the treatment of inflammatory bowel disease: an investigation using a mouse gut microbiome and metabolomics model

**DOI:** 10.3389/fcimb.2026.1830455

**Published:** 2026-06-10

**Authors:** Jiayin Lu, Shengnan Bi, Yingchun Gao, Yiyue Zhang, Tong Zhang, Mengmeng Sun, Min He, Xinhua Chen, Bo Yang

**Affiliations:** 1 College of Acupuncture and Tuina, Changchun University of Chinese Medicine, Changchun, Jilin, China; 2 Department of Pharmacy, Affiliated Hospital of Changchun University of Chinese Medicine, Changchun, Jilin, China; 3 Department of Quality Control, Affiliated Hospital of Changchun University of Traditional Chinese Medicine, Changchun, Jilin, China; 4 Northeast Asia Research Institute of Traditional Chinese Medicine, Changchun University of Chinese Medicine, Changchun, Jilin, China; 5 Department of Acupuncture and Tuina, Affiliated Hospital of Changchun University of Chinese Medicine, Changchun, Jilin, China; 6 Research Center of Traditional Chinese Medicine, Gansu, Lanzhou, China

**Keywords:** acupoint, electroacupuncture, gut microbiome, inflammatory bowel disease, metabolomics

## Abstract

Electroacupuncture (EA) serves as an effective complementary therapy in the treatment of inflammatory bowel disease (IBD) within traditional Chinese medicine, with the therapeutic efficacy significantly influenced by the selection of specific acupoints. However, the regulatory mechanisms by which certain acupoints exhibit therapeutic benefits in IBD remain poorly understood. The research project intends to explore the mechanisms of action and regulatory variations in the treatment of IBD by the points Dachangshu (BL25), Tianshu (ST25), and Shangjuxu (ST37). This study used a sodium dextran sulfate-induced IBD mouse model to assess the therapeutic efficacy of three acupoints by evaluating body weight, colon morphology, inflammatory cytokines, gut microbiota, and metabolites. The results indicate that electroacupuncture of these three acupoints did not significantly alter IL-1β levels, but all three acupoints reduced IL-6 levels, with the most significant reduction observed at the Dachangshu acupoint. Furthermore, electroacupuncture treatment improved the structural characteristics of intestinal tissue and slowed the rate of weight loss. In IBD mice, electroacupuncture of three acupoints was associated with significant alterations in the intestinal microbiota and metabolic pathways. The majority of the metabolic pathways involved by these three acupoints are related to lipid, amino acid, and energy metabolism. Additionally, different acupuncture stimulation points also exhibited their own unique metabolic characteristics. These findings provide preliminary correlative evidence for the clinical application of acupuncture point selection in the treatment of IBD.

## 1 Introduction

Inflammatory bowel disease (IBD) is a chronic, immune-mediated disorder characterized by relapsing inflammation of the gastrointestinal tract. IBD principally comprises two clinical subtypes: Crohn’s disease (CD) and ulcerative colitis (UC) (Singh and Bernstein, 2022). The pathogenesis of IBD is multifactorial and complex, involving genetic susceptibility, environmental exposures, intestinal microbiota, immune dysregulation, and additional risk determinants (Agrawal et al., 2022). IBD prevalence is rising worldwide, shifting from a Western to a global public health issue, driven in developing regions by socioeconomic changes such as Westernized lifestyles, diet, and hygiene (Catalan-Serra and Sebastian, 2025; Ng, 2014). IBD can occur at any age, most commonly in adolescence and early adulthood, but also in infancy and early childhood. Treatment is time-consuming and costly in adults, while care and long-term management are more complex in children (Vuijk et al., 2024; Al-Beltagi et al., 2025).

IBD primarily presents with gastrointestinal symptoms, including diarrhea, abdominal pain or discomfort, and hematochezia, accompanied by systemic manifestations such as fever, fatigue, weight loss, and night sweats. Ulcerative colitis typically originates in the colon, whereas Crohn’s disease can affect any segment of the gastrointestinal tract (Davis and Kellerman, 2022). Beyond intestinal involvement, IBD often presents with extraintestinal manifestations affecting the skin, musculoskeletal system, eyes, and hepatobiliary tract, and it also has a considerable impact on patient mental health (Gupta and Choi, 2025). Symptoms of anxiety and depression are common in individuals with IBD, with approximately one-third experiencing anxiety and one-quarter experiencing depression (Barberio et al., 2021).

The pathophysiology of IBD is complex and involves inappropriate activation of the mucosal immune system in genetically susceptible individuals, driven by dysregulated interactions between the host, the gut microbiota, and environmental factors (Wan et al., 2025). Gut microorganisms are increasingly recognized as potential biomarkers for IBD, and dysbiosis of the gut microbiota can heighten immune responses, disrupt intestinal barrier integrity, and promote disease development (Qiu et al., 2022; Chen and Wang, 2022; Wang et al., 2024a). Experimental and clinical studies show increased abundance or activity of bacterial groups such as Enterobacteriaceae, Clostridium spp., and Enterococcus spp., together with reduced beneficial commensals and overall microbial diversity, changes that are thought to contribute to the initiation and progression of IBD (Iliev et al., 2025; Haneishi et al., 2023).

Although the gut microbiota is known to contribute to the pathophysiology of IBD, the precise mechanisms driving intestinal inflammation remain incompletely understood. Gut microbial activity is closely linked to metabolites such as lipids, amino acids, and organic acids, which modulate host immunity and metabolism and contribute to the onset and progression of IBD (Vich Vila et al., 2024). Microbial metabolites act on multiple immune cell types (including T cells, B cells, dendritic cells, and macrophages), are implicated in immune-mediated inflammatory diseases such as IBD, and represent key targets for elucidating disease mechanisms and developing diagnostic and therapeutic strategies (Yang and Cong, 2021).

Current IBD management primarily relies on individualized pharmacotherapy, typically involving the use of mesalazine, corticosteroids, and antibiotics, often in combination with immunosuppressants and biologic agents. However, drug therapy alone is often insufficient, and a proportion of patients ultimately require surgical intervention (Triantafillidis et al., 2024). Emerging microbiome- and cell-based approaches, including stem cell therapy, organoid-derived intestinal tissue regeneration, and fecal microbiota transplantation (FMT), show promise for restoring intestinal barrier function and modifying disease course in IBD (Villablanca et al., 2022; Bethlehem et al., 2024).

From a clinical perspective, IBD is often managed with combination therapy, using multiple drugs or integrating pharmacologic and nonpharmacologic approaches, which can provide significant benefit in selected patients (Dai et al., 2023). Traditional Chinese medicine, particularly acupuncture, is widely used as a complementary therapy in IBD and is thought to exert benefit by modulating gut immunity and inflammation, sensory signaling, neuroendocrine-immune networks, the intestinal microbiota, and the brain-gut axis (Cheifetz et al., 2017; Liu et al., 2024). Electroacupuncture involves inserting fine needles into acupoints and delivering controlled electrical stimulation via an external device, making it easier to standardize and potentially more consistent in its therapeutic effects than manual acupuncture (Song et al., 2019).

The mechanisms underlying the therapeutic effects of electroacupuncture in IBD are increasingly being elucidated. Electroacupuncture has been shown to regulate the Piezo1-mitochondria-iron phosphatase axis, thereby preserving mitochondrial function and redox homeostasis, reducing colonic epithelial damage, and preventing iron loss in intestinal epithelial cells in IBD (He et al., 2025). Experimental evidence suggests that electroacupuncture can alleviate abdominal pain in IBD by mitigating TNBS-induced intestinal inflammation, likely through the enhancement of endogenous cannabinoids and CB2 receptor signaling, which reduces macrophage activity, suppresses inflammatory cytokine production, and decreases visceral hypersensitivity (Zhang et al., 2022). Studies in spinal cord injury mouse models show that electroacupuncture promotes recovery of intestinal transit and restores colonic morphology, while also markedly reshaping dysbiotic gut microbiota characterized by increased Proteobacteria, Clostridia, Bacillales, and Dorella (Cheng et al., 2022). Collectively, accumulating evidence indicates that electroacupuncture exerts therapeutic effects in IBD by modulating colonic macrophage polarization, repairing the intestinal barrier, and reshaping the gut microbiota (Xu et al., 2023; Yang et al., 2024a; Liu et al., 2022).

The therapeutic efficacy of electroacupuncture in IBD is closely linked to acupoint selection, yet it remains unclear how specific acupoints influence treatment outcomes or whether they act through shared or distinct mechanisms. Clarifying how specific acupoints shape the therapeutic effects of electroacupuncture in IBD, and whether they act through shared or distinct mechanisms, is therefore crucial for optimizing acupuncture-based treatment.

## 2 Methods

### 2.1 Animals

Laboratory mice were obtained from Beijing Viton Lihua Biotechnology Co., Ltd. (Beijing, China). A total of 60 healthy male C57BL/6 mice weighing 22 ± 2 g were used. Mice were housed in standard cages under controlled conditions (22 °C; relative humidity 40-60%). Animals had free access to food and water and were acclimatized for one week before the experiments. All procedures complied with established guidelines for the care and use of laboratory animals. The experimental protocol was approved by the Animal Ethics Committee of Changchun University of Chinese Medicine (approval No. 2023162). Every effort was made to minimize animal suffering throughout the study.

### 2.2 Induction of IBD model

Based on a review of methodological studies on IBD animal models, we aimed to establish a chronic, low-grade intestinal inflammation model in mice that mimics the prolonged course of human IBD. This approach avoids the high morbidity and mortality of acute colitis models and allows for subsequent electroacupuncture interventions. We therefore conducted preliminary experiments to optimize and refine the modeling protocol (Wirtz et al., 2007; Yang et al., 2024b). In this study, we used dextran sodium sulfate (DSS), a simple and effective chemical induction method, to generate an IBD mouse model. DSS powder was dissolved in ultrapure water to prepare a 0.75% solution, which was supplied as the animals daily drinking water. After 14 days of DSS administration, the IBD model was successfully established.

### 2.3 Grouping and treatment

**Figure 1:**
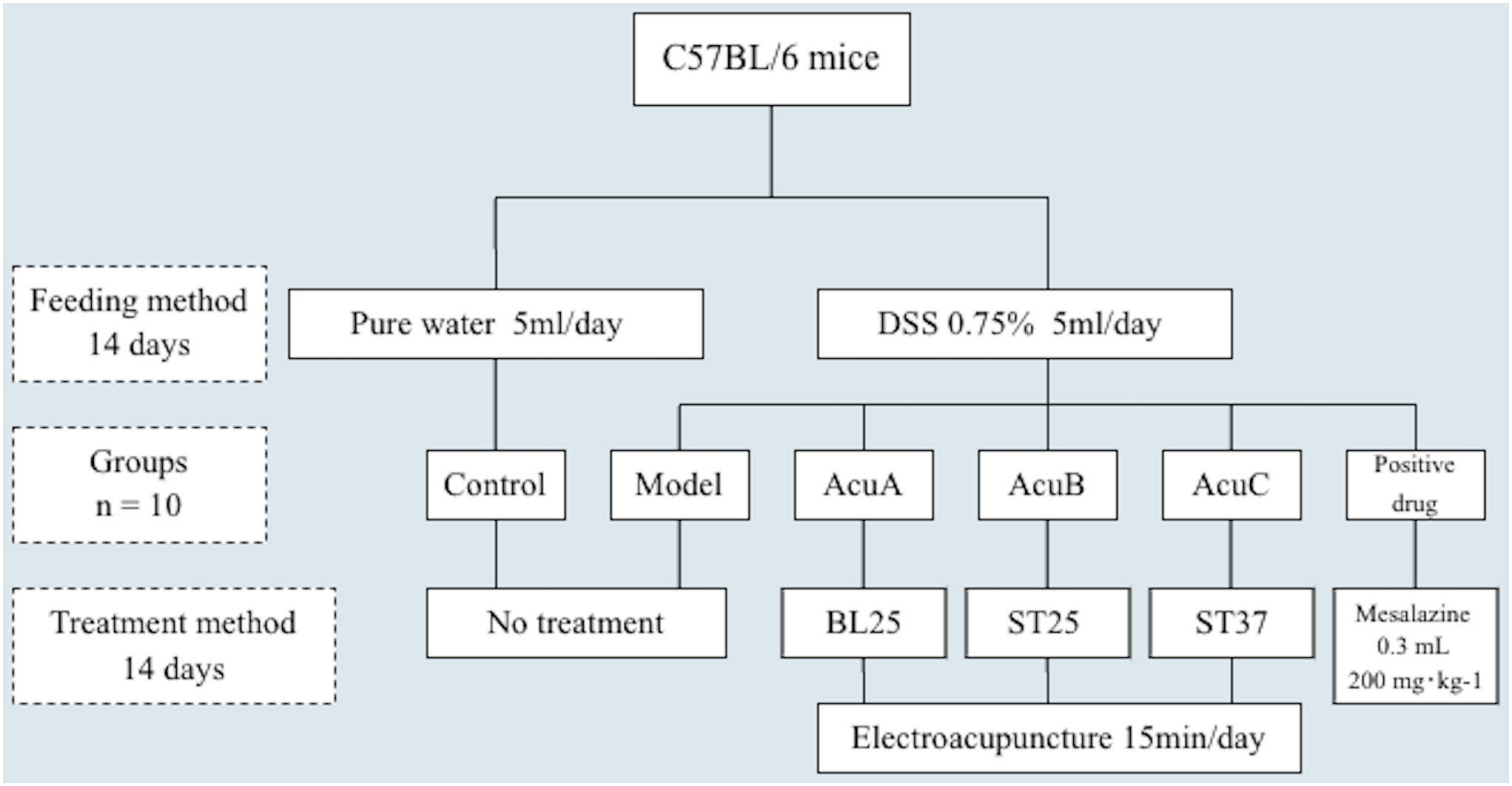
The mice information was grouped and corresponding intervention methods were adopted. The six groups of mouse models were respectively named as Control, Model, AcuA, AcuB, AcuC, and Positive drug. Acupoints: Dachangshu (BL25); Tianshu (ST25); Shangjuxu (ST37).

After one week of acclimatization, the body weights of all 60 mice were recorded. Mice were then randomly assigned, using a random-number table, to six groups: Control (CTRL), Model (Model), Dachangshu (AcuA), Tianshu (AcuB), Shangjuxu (AcuC), and Positive drug (Positive Drug). The detailed grouping scheme is shown in **Figure 1**. Mice in the control group received a standard diet and tap water. Mice in the, AcuA, AcuB, AcuC, and Positive Drug groups were given 0.75% DSS in their drinking water.

The Dachangshu point (BL25, bilateral) is located about 5 mm lateral to the spinous process of the fourth lumbar vertebra. It is the Back-Shu point of the Hand-Yangming Large Intestine Meridian. The Tianshu point (ST25, bilateral) is about 5 millimeters on either side of the navel and acts as the Mu point of the Hand-Yangming Large Intestine Meridian. The Shangjuxu point (ST37, bilateral) is located on the mouse’s hindlimb, approximately 6 millimeters below the Zusanli point (ST36). It is the lower convergence point of the Hand-Yangming Large Intestine Meridian. Mice were fastened to specially designed mouse plates before treatment to prevent them from escaping.

Subsequently, all six groups of mice were anesthetized with isoflurane inhalation. The three electroacupuncture groups were treated using disposable 1.0-inch needles (0.25 mm × 13 mm; Huatuo, Anhui, China). Needles were inserted perpendicularly and slowly into the bilateral acupoints to a depth of approximately 3 mm. The needle handles were then connected to an electroacupuncture stimulator (Huatuo SDZ-V) set to a sparse-dense waveform at 2 Hz. Stimulation intensity was adjusted until slight twitching of the limbs was observed. Electroacupuncture was administered once daily for 15 min over 14 consecutive days. During the treatment period, mice in the positive drug group received oral mesalazine once daily at 10:00 a.m. Mesalazine was administered at 200 mg·kg^-^¹ in a volume of 0.3 mL for 14 days.

### 2.4 Sample collection

Throughout induction and treatment, mouse body weight was monitored regularly. After the final treatment, mice were fasted for 12 h. All animals were then euthanized for sample collection. Colon tissue was harvested for histological examination, and blood was collected for biochemical analyses. In addition, fecal samples were collected and placed in cryogenic tubes. Samples were snap-frozen in liquid nitrogen and stored at −80 °C for subsequent 16S rDNA sequencing and metabolomic analyses.

### 2.5 Body weight and colon histology

Body weight was measured every other day over a 10-day period, and the general health status of the mice was carefully observed. Colon tissue samples were collected, rinsed several times with ice-cold physiological saline, fixed in 4% formaldehyde, dehydrated through a graded ethanol series, embedded in paraffin, sectioned, and mounted on slides. Sections were dewaxed, stained with hematoxylin and eosin (H&E), dehydrated, cleared, and coverslipped. Finally, stained sections were examined under a light microscope.

### 2.6 ELISA for inflammatory factors

Enzyme-linked immunosorbent assay (ELISA) was used to quantify inflammatory cytokines in mouse serum. The levels of interleukin-6 (IL-6) and interleukin-1β (IL-1β) were determined. Briefly, standards were serially diluted, and serum samples were added to the wells. Plates were sealed and incubated at 37 °C for 30 min. After the addition of the enzyme conjugate and further incubation, the plates were washed. Subsequently, 50 μL of chromogen A and 50 μL of chromogen B were added to each well, and the plates were incubated in the dark at 37 °C for 10 min before the reaction was terminated.

### 2.7 DNA extraction and 16S rDNA sequencing

On the final day of the experiment, one to two fresh fecal pellets were collected from each mouse for analysis. In total, 42 fecal samples (n = 7 per group) were obtained for DNA extraction and 16S rDNA sequencing. Fecal samples were immediately frozen and stored at −80 °C. Total genomic DNA was extracted using a commercial kit (Magen, D6356-02, Guangzhou, China) according to the manufacturer instructions. DNA quality and quantity were assessed by agarose gel electrophoresis and a NanoDrop spectrophotometer (Thermo Fisher Scientific, Waltham, MA, USA). Extracted DNA was diluted to 1 ng/μL and stored at −20 °C until further use. Using the diluted DNA as a template, the bacterial 16S rRNA gene was amplified by PCR with barcoded primers and Ex Taq reagents (Takara, Shiga, Japan). Universal primers 343F and 798R were used to amplify the V3-V4 region of the 16S rRNA gene for bacterial diversity analysis.

### 2.8 GC-MS metabolomics

Fecal samples were homogenized by ultrasonic disruption and centrifuged to separate their components. The supernatant was removed, the remaining solution was evaporated to dryness, and an oxidation reaction was performed. Subsequently, 50 μL of BSTFA, 20 μL of n-hexane, and 10 μL of a mixed internal standard were added, and the mixture was vortexed and incubated at 70 °C for 60 min. Metabolomic profiling was performed using gas chromatography-mass spectrometry (GC-MS). Raw GC-MS data files were converted from D to ABF format using dedicated file-conversion software to facilitate data processing. The converted data were then processed and analyzed with MS-DIAL software to generate the final data matrix.

### 2.9 LC-MS metabolomics

The intestinal contents of fecal samples were homogenized with internal standards, extracted by ultrasonication, centrifuged, and the supernatants were transferred to liquid chromatography-mass spectrometry (LC-MS) vials. After drying, residues were reconstituted in 300 μL of methanol-water (1:4), allowed to stand, and centrifuged again. The resulting supernatants were filtered, transferred to sample vials, stored at −80 °C, and subsequently analyzed by LC-MS. Raw LC-MS data were processed using Progenesis QI v2.3 (Nonlinear Dynamics, Newcastle, UK) to generate a data matrix for downstream analysis.

### 2.10 Statistical analysis

Statistical analyses were performed using GraphPad Prism 10.0. For normally distributed data, as assessed by the Shapiro-Wilk test (p > 0.05), between-group comparisons were conducted using independent-samples t-tests (two groups) or one-way analysis of variance (ANOVA; multiple groups). When group sizes were equal, Tukey *post hoc* test was applied; when unequal, the Tukey-Kramer test was used. Non-normally distributed data are presented as median and interquartile range (IQR). For non-parametric comparisons between two groups, the Wilcoxon rank-sum test was used. For non-parametric comparisons among multiple groups, the Kruskal-Wallis H test was applied. All tests were two-tailed, and p < 0.05 was considered statistically significant.

Principal component analysis (PCA), orthogonal partial least squares-discriminant analysis (OPLS-DA), and partial least squares-discriminant analysis (PLS-DA) were conducted for microbiome and metabolome data by importing the matrices into R programming language. Validation parameters for the OPLS-DA s (R²Y, Q², and permutation test results) are summarized in **Supplementary Table 1**. Variable importance in projection (VIP) scores were calculated to assess the contribution of each variable to group separation. Subsequently, two-tailed Student’s t-tests were used to evaluate the statistical significance of metabolic differences between groups. Metabolites with VIP > 1.0 and p < 0.05 were considered differentially expressed.

This study used a blinded design for all outcome assessments. Because electroacupuncture requires specialized expertise and the anatomical locations of the acupoints differ markedly, the practitioners delivering the interventions were aware of group allocation. Body weight measurement, colonic length measurement, histopathological scoring, ELISA, 16S rRNA sequencing, metabolomic analysis, and statistical analysis were all performed by investigators blinded to group allocation.

## 3 Results

### 3.1 Electroacupuncture reduces weight loss and colon damage in IBD mice

**Figure 2:**
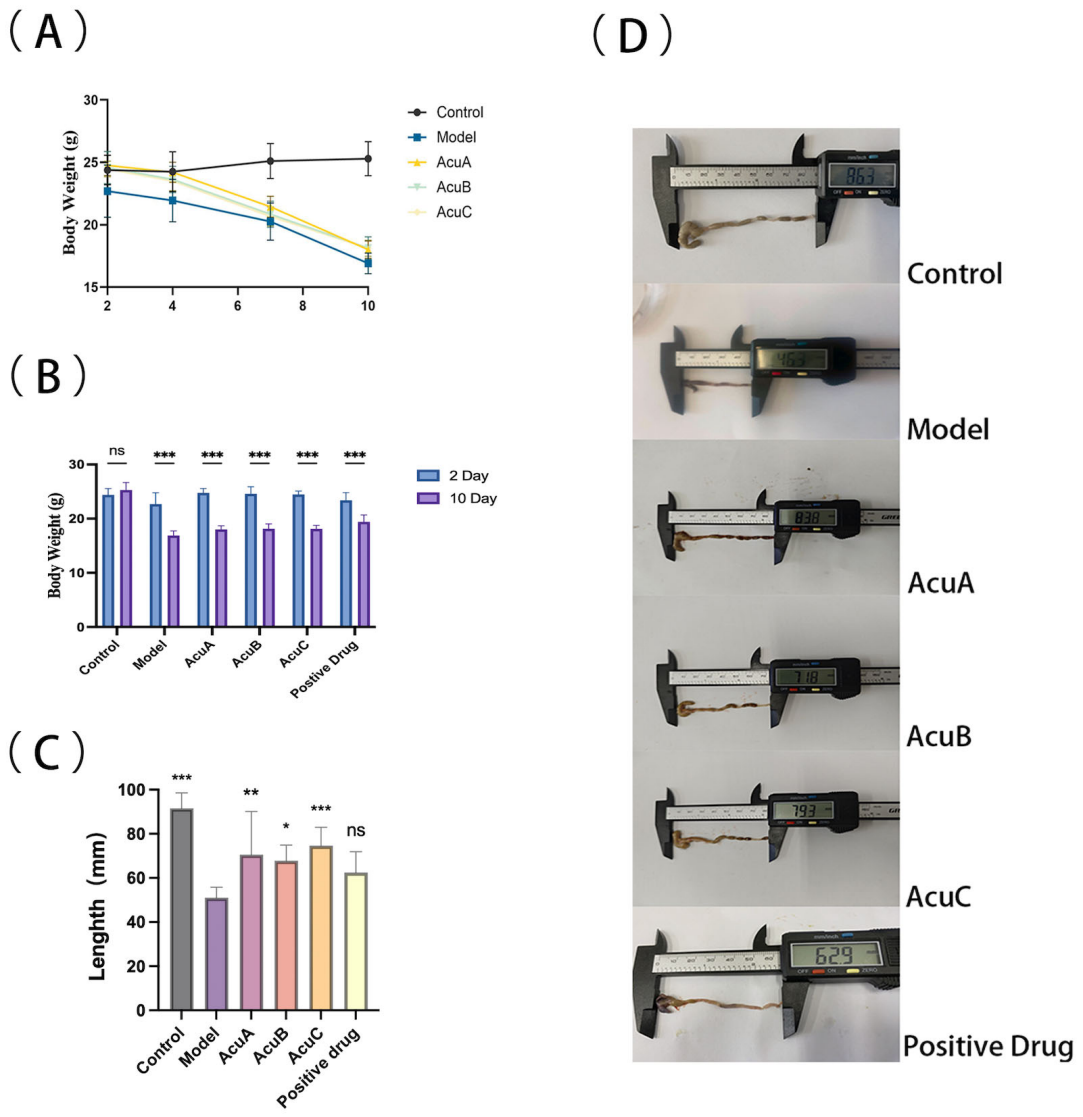
The body weight of each group of mice and the overall structure of their colons (n = 10 per group). **(A)** Mouse weights of each group over a period of 10 consecutive days. **(B)** Mouse weights at the beginning and end of the experiment. **(C)** Compare with the colon length of the model group. **(D)** Colon morphology. Significance levels: *p < 0.05, **p < 0.01, ***p < 0.001, ns, not significant (p ≥ 0.05).

Inflammatory bowel disease can cause malnutrition and weight loss, so body weight is commonly used as a general indicator of disease severity and recovery in experimental IBD mouse model (Jabłońska and Mrowiec, 2023). As shown in **Figure 2A**, mice in the control group maintained relatively stable body weight over the 10-day observation period. In contrast, the model group showed substantial weight loss over the same period. Weight loss in the three electroacupuncture groups and the positive drug group progressed more slowly than in the model group. As illustrated in **Figure 2B**, comparison of body weight before and after treatment showed that the model group had the largest change in body weight among all groups. Mice receiving active treatments showed varying degrees of attenuation of weight loss compared with the model group.

Electroacupuncture attenuated colon shortening and pathological changes in mice with inflammatory bowel disease. As shown in **Figure 2C**, colon length was significantly shorter in the model group than in the control group (p < 0.05). After treatment, the positive drug group showed a trend toward increased colon length, although this did not reach statistical significance. In contrast, all three electroacupuncture groups had significantly longer colons than the model group (p < 0.05), with the Shangjuxu acupoint having the strongest impact.

As shown in **Figure 2D**, intestines from control mice appeared smooth, with only a small amount of mucus on the mucosal surface. The mucosal folds showed no erosion, ulceration, or bleeding, and the luminal contents were uniformly granular. In contrast, the model group exhibited a markedly shorter average colon length than the control group, with pronounced mucosal congestion, edema, erosion, and ulceration. The positive drug group and all three electroacupuncture groups showed better overall mucosal appearance than the model group. Despite mild edema on the mucosal surface, the musculature beneath the mucosal folds remained clearly visible. Compared with the model group, treated mice showed markedly less colonic mucosal hyperemia and erosion.

**Figure 3:**
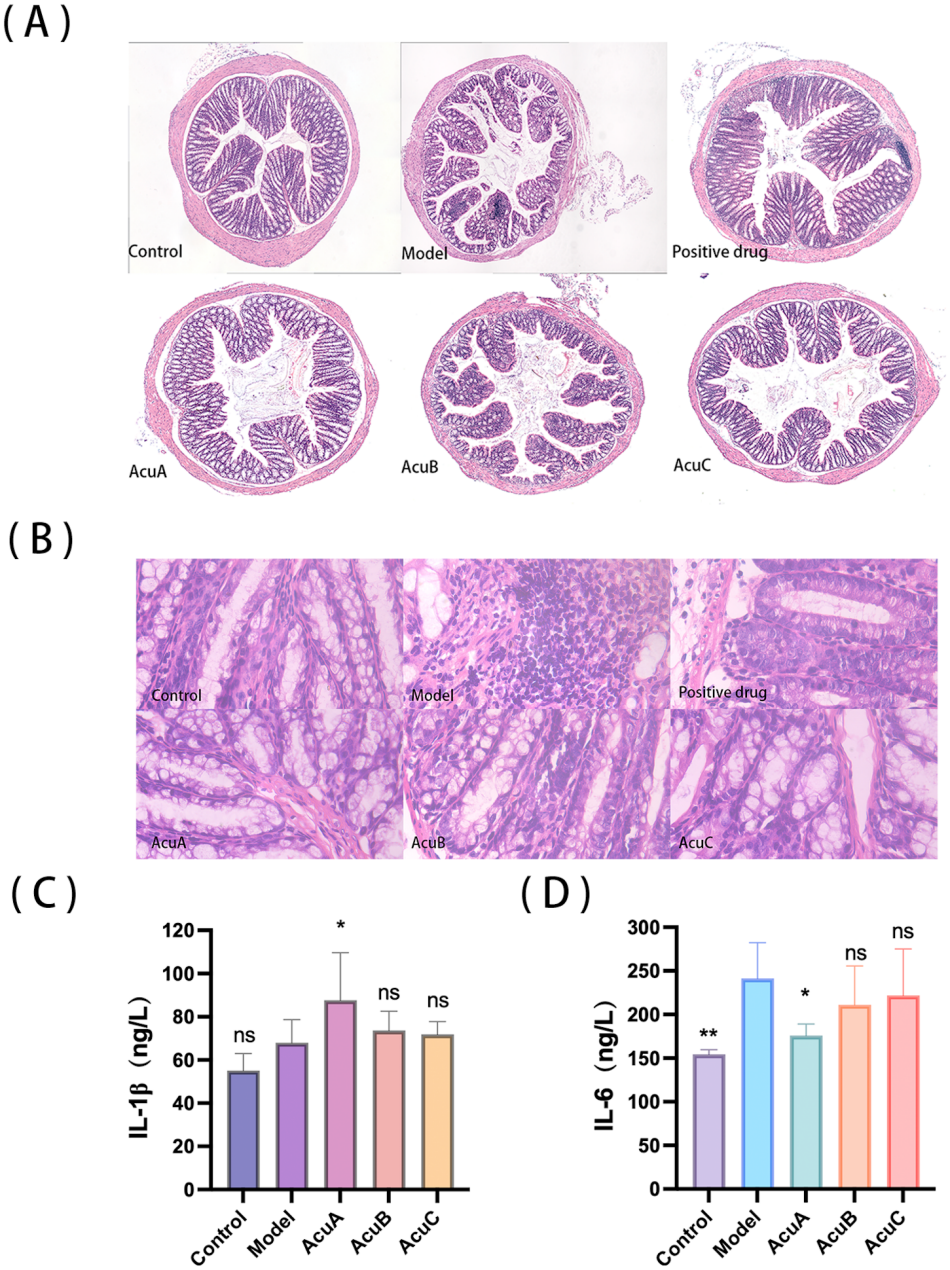
The colon tissues and inflammatory factors of each group of mice were compared (n = 10 per group). **(A)** Hematoxylin-eosin staining (×100). **(B)** Hematoxylin-eosin staining (×400). **(C)** Comparison with the IL-1β level of the model group. **(D)** Comparison with the IL-6 level of the model group. Significance levels: *p < 0.05, **p < 0.01, ***p < 0.001, ns, not significant (p ≥ 0.05).

To assess the effects of electroacupuncture at different acupoints on microscopic pathology of the colon, we examined hematoxylin-eosin-stained colonic tissues. As shown in **Figure 3A**, the control group displayed an intact colonic mucosal epithelium with regularly arranged glands. In contrast, the model group exhibited mucosal injury with reduced gland number and architectural distortion. Colonic crypts were shortened, consistent with epithelial hypoplasia, with localized congestion and edema. The positive drug group showed milder glandular damage with reduced bleeding and ulceration. All three electroacupuncture groups displayed less morphological damage of the colonic mucosa. At higher magnification (**Figure 3B**), the control group showed no inflammatory cell infiltration, with orderly cellular architecture. The model group demonstrated marked inflammatory cell infiltration. The positive drug group showed reduced inflammatory cell infiltration. The three electroacupuncture groups exhibited minimal inflammatory cell infiltration.

### 3.2 Electroacupuncture regulates inflammatory cytokines in IBD mice

Key interleukins IL-1β and IL-6, central mediators of inflammation, were measured in mouse blood samples (Aebisher et al., 2024). As shown in **Figure 3C**; IL-1β levels were higher in the model group than in controls, although the difference was not statistically significant. Electroacupuncture at Tianshu and Shangjuxu was associated with lower IL-1β levels. Across groups, IL-6 levels were higher in the model group than in controls (**Figure 3D**). Mice receiving electroacupuncture showed reduced IL-6 levels, with a significant reduction at the Dachangshu group compared with the model group.

### 3.3 Electroacupuncture at different acupoints alters gut microbiota composition

**Figure 4:**
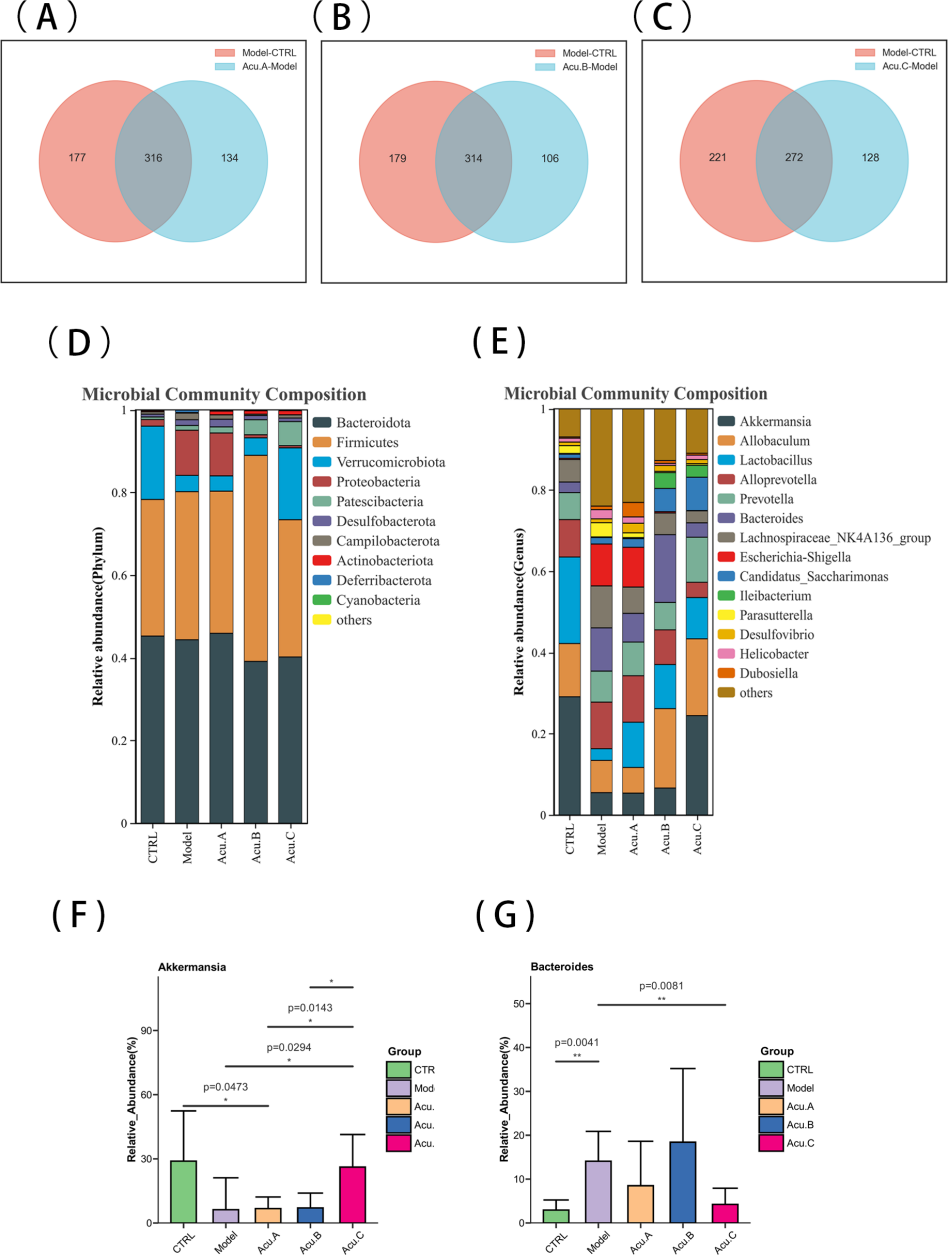
Total number and composition of each group of bacterial communities. **(A–C)** Venn diagrams showing the number of ASVs in each group. **(D)** Structural composition of bacterial communities at the phylum level in each group. **(E)** Structural composition of bacterial communities at the genus level in each group. **(F)** Comparison of Akkemansia in each group. **(G)** Comparison of Bacteroides in each group.

Reduced gut microbiota abundance and diversity are closely associated with the development of inflammatory bowel disease (Weingarden and Vaughn, 2017). To evaluate whether different electroacupuncture acupoint selections exert distinct regulatory effects on gut microbiota quantity and composition, we used 16S rRNA sequencing to assess overall microbiota abundance across groups (Wang et al., 2024b). **Figures 4A–C** present statistical analyses of amplicon sequence variants (ASVs). A total of 134 ASVs were assigned to the Dachangshu group, 106 ASVs to the Tianshu group, and 128 ASVs to the Shangjuxu group. Acupoint selection influenced the total number of ASVs, and electroacupuncture at Dachangshu produced a greater change than at Tianshu or Shangjuxu.

Gut microbiota composition was analyzed to assess community diversity at the phylum and genus levels in each group. The results revealed marked differences in microbiota abundance between the model and control groups, and differential regulatory effects on the microbial community among acupoint treatments. At the phylum level, Bacteroidetes, Firmicutes, Verrucomicrobiota, and Proteobacteria predominated (**Figure 4D**). In the IBD model group, Verrucomicrobiota decreased, whereas Proteobacteria increased. Dachangshu more strongly were associated with changes in Bacteroidetes abundance, whereas Shangjuxu had a greater impact on Verrucomicrobiota and Proteobacteria. At the genus level (**Figure 4E**), Akkermansia, Allobaculum, Lactobacillus, Alloprevotella, Prevotella, and Bacteroides dominated across groups. The IBD model group showed reduced Akkermansia and Lactobacillus with increased Bacteroides. The Shangjuxu group exhibited a microbial profile more similar to the control group. Compared with the model group, Shangjuxu notably were associated with changes in Akkermansia and Bacteroides abundances (**Figures 4F, G**).

**Figure 5:**
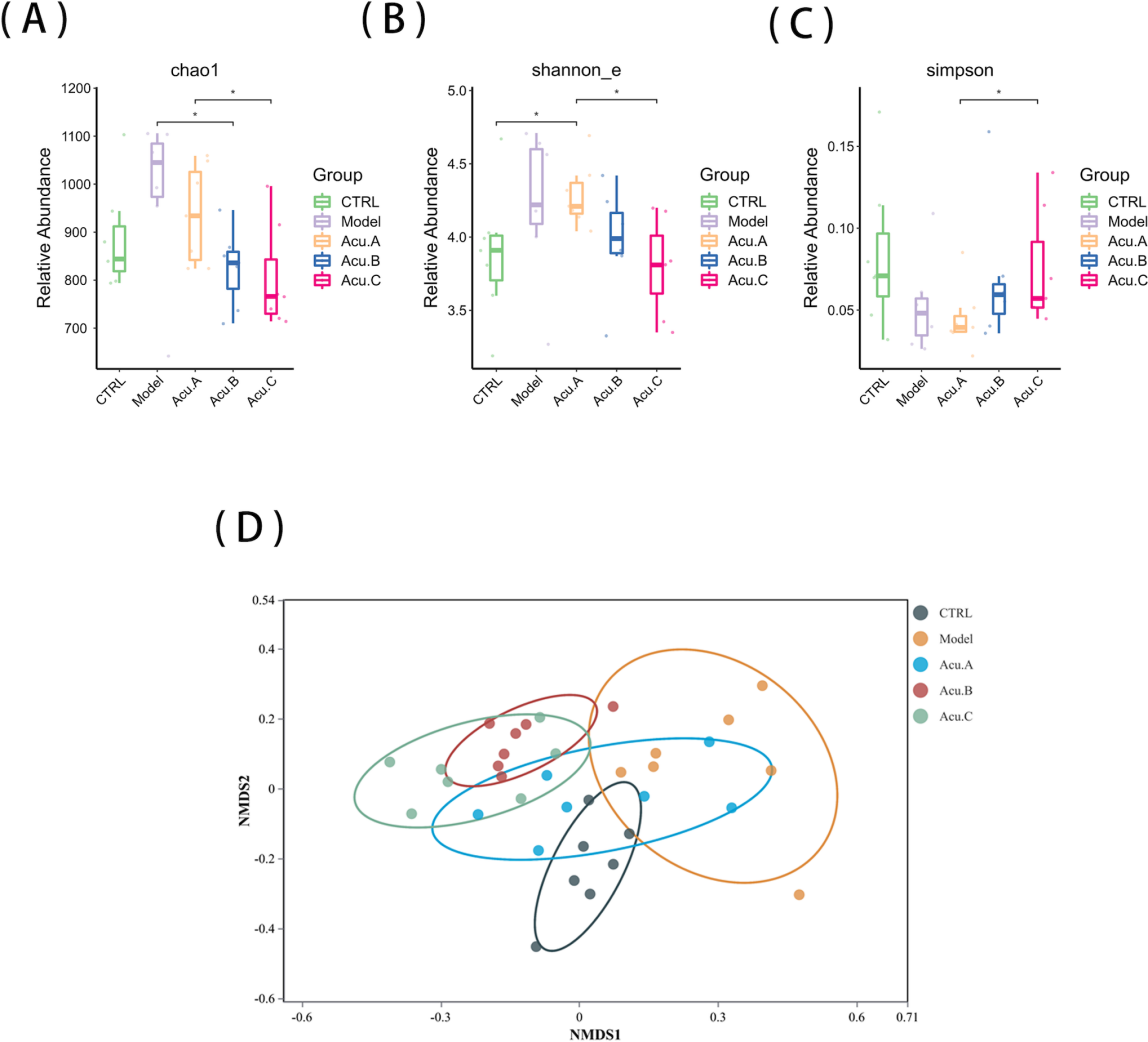
α analysis and β analysis. **(A)** Comparison of chao1 index among all groups. **(B)** Comparison of shannon index among all groups. **(C)** Comparison of simpson index among all groups. **(D)** Comparison of NMDS analysis among all groups.

The α-diversity was compared using the Chao1, Shannon, and Simpson indices. Although the model group showed higher Chao1 and Shannon indices than controls, among the three electroacupuncture groups, the Shangjuxu group most closely resembled controls on these indices (**Figures 5A, B**). The Simpson index was significantly lower in the model group and significantly higher in the Shangjuxu group (**Figure 5C**).

Non-metric multidimensional scaling (NMDS), a β-diversity approach, was used to visualize sample clustering. NMDS plots (**Figure 5D**) showed clear separation between the model and control groups, indicating a substantial impact of IBD on overall gut microbiota structure. The model group also differed from all three electroacupuncture groups, indicating that electroacupuncture altered the microbial community composition. Among the three acupoint groups, Dachangshu was farthest from the model group and closest to the control group in NMDS space. These patterns suggest that the Dachangshu group microbial community most closely resembled controls and differed most from the model group.

**Figure 6:**
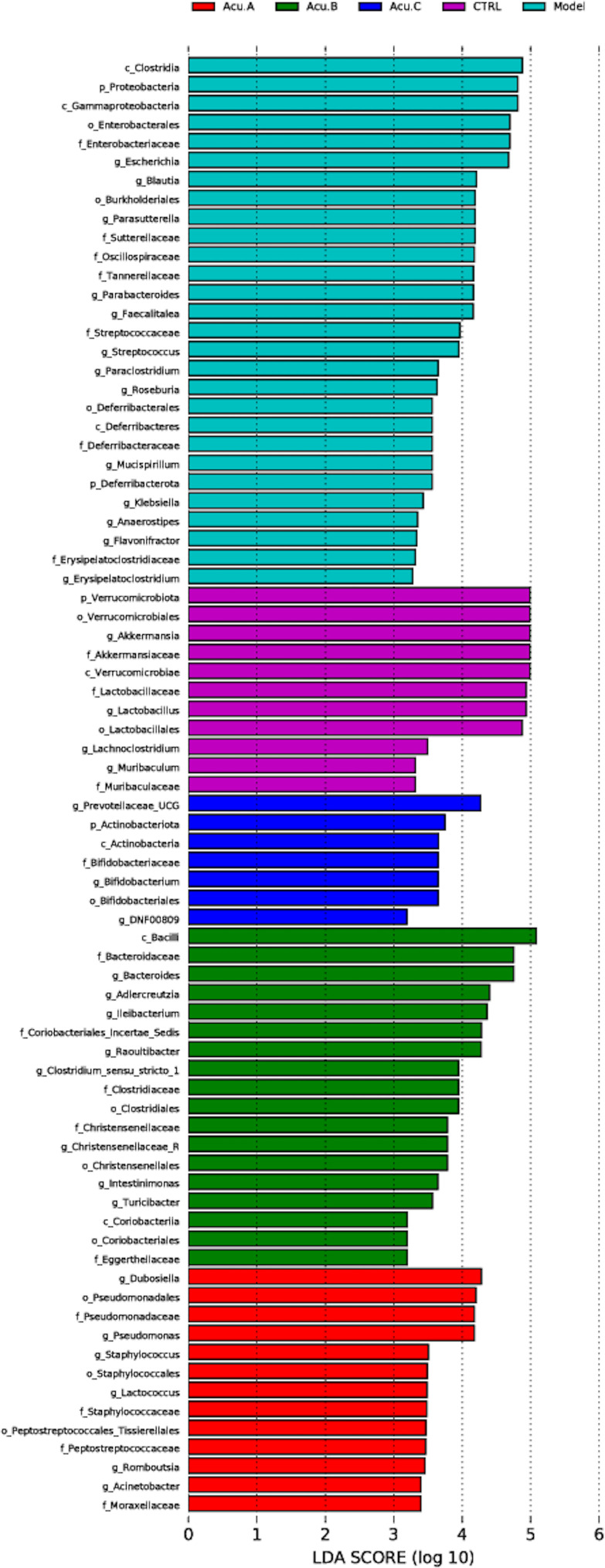
The LDA scores of several abundant species in the fecal microbiota of Control, Model, Acu.A, Acu.B, and Acu.C.

To better characterize post-treatment shifts in fecal microbiota composition, high-dimensional clustering was performed using Linear Discriminant Analysis Effect Size (LEfSe). Only taxa with LDA score > 2 and p < 0.05 (Wilcoxon rank-sum test) are shown. The histogram displays LDA scores (log10 transformed). As shown in **Figure 6**, the model group differed markedly from controls, and distinct bacterial taxa were differentially abundant across the acupoint electroacupuncture groups. The Dachangshu group showed higher abundances of Dubosiella, Pseudomonadales, Pseudomonadaceae, and Pseudomonas. The Tianshu group was enriched in Bacilli, Bacteroidaceae, and Bacteroides. The Shangjuxu group exhibited higher levels of Prevotellaceae_UCG and Actinobacteriota.

### 3.4 Electroacupuncture at different acupoints alters metabolites and metabolic pathways

Numerous studies indicate that alterations in the gut microbiota and metabolome are closely associated with the course of inflammatory bowel disease (Franzosa et al., 2019). In IBD, disruption of the intestinal barrier permits microbial translocation into the submucosa, thereby affecting host metabolism (Upadhyay et al., 2023). Metabolic shifts reflect dysbiosis and its effects on host immune responses and metabolic pathways (Verdugo-Meza et al., 2020). In our previous work, we observed that three acupoints were associated with distinct alterations in the intestinal microbiota of IBD mice. To further delineate these differences, we performed comprehensive GC-MS and LC-MS analyses of colonic contents from IBD mice to compare metabolite profiles and metabolic pathways across the three acupoint groups.

**Figure 7:**
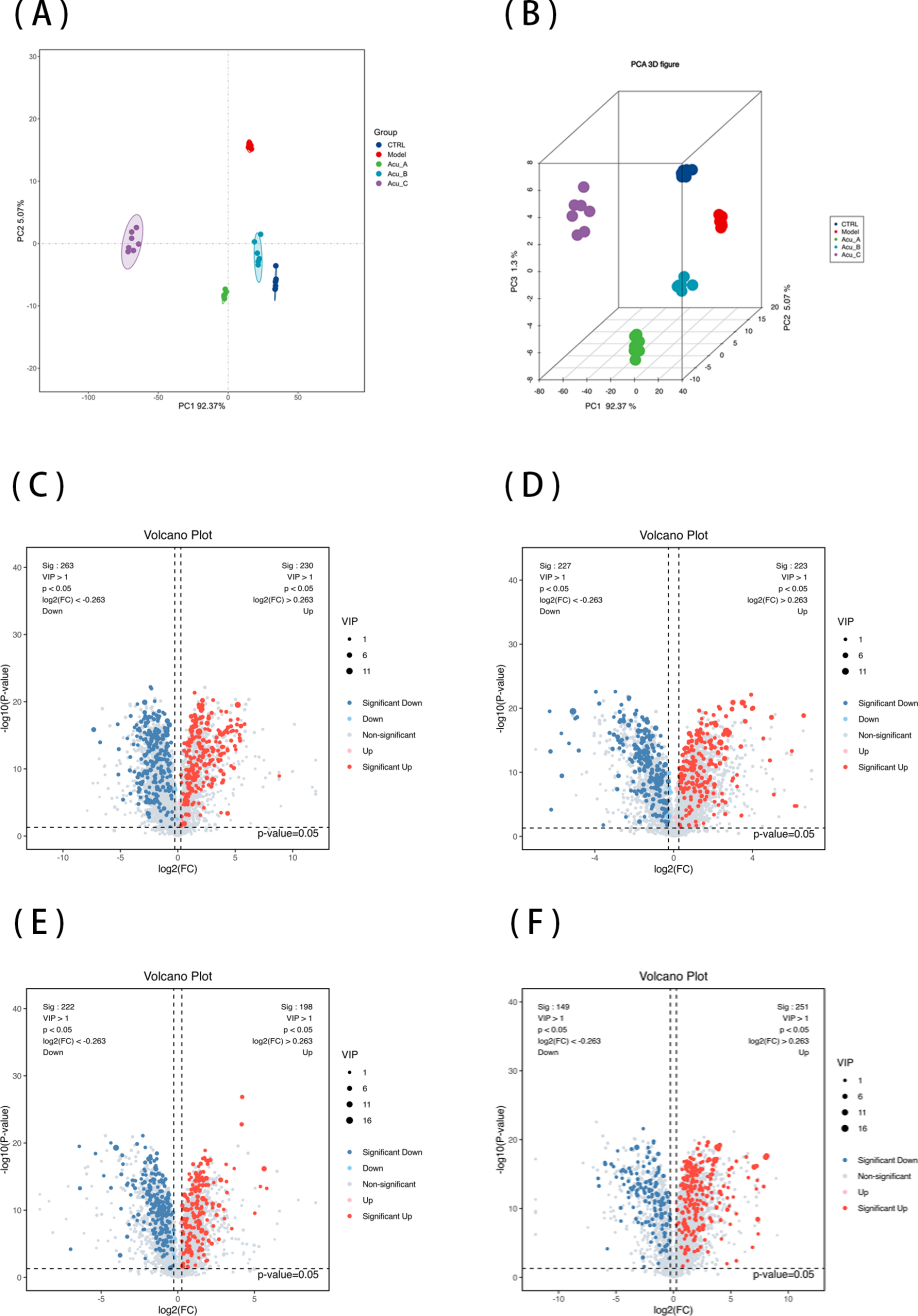
Principal component analysis and volcano plot of differential metabolites of the colon contents in each group of mice. **(A)** and **(B)** are the principal component analysis diagrams of the control group, model group, and acupuncture group in GC-MS and LC-MS. **(C)** is the volcano plot comparing Model vs CTRL for differential metabolites. **(D)** is the volcano plot comparing Acu.A vs Model for differential metabolites. **(E)** is the volcano plot comparing Acu.B vs Model for differential metabolites. **(F)** is the volcano plot comparing Acu.C vs Model for differential metabolites. Red indicates an increase, while blue indicates a decrease.

Principal component analyses (**Figures 7A, B**) showed significant differences in colonic metabolites between control and model groups in both GC-MS and LC-MS datasets. These findings indicate an altered metabolic profile in IBD mice. All three electroacupuncture groups differed from the model group in colonic metabolites. Thus, electroacupuncture at each acupoint can modulate metabolic abnormalities in IBD mice.

We next examined changes in fecal metabolites in IBD model mice following treatment at three acupoints. In Model vs CTRL, 493 metabolites were differentially expressed, with 230 significantly upregulated and 263 significantly downregulated (**Figure 7C**). In AcuA vs Model, 450 metabolites were differentially expressed (223 upregulated, 227 downregulated) (**Figure 7D**). In AcuB vs Model, 420 metabolites were differentially expressed (198 upregulated, 222 downregulated) (**Figure 7E**). In AcuC vs Model, 400 metabolites were differentially expressed (251 upregulated, 149 downregulated) (**Figure 7F**).

**Figure 8:**
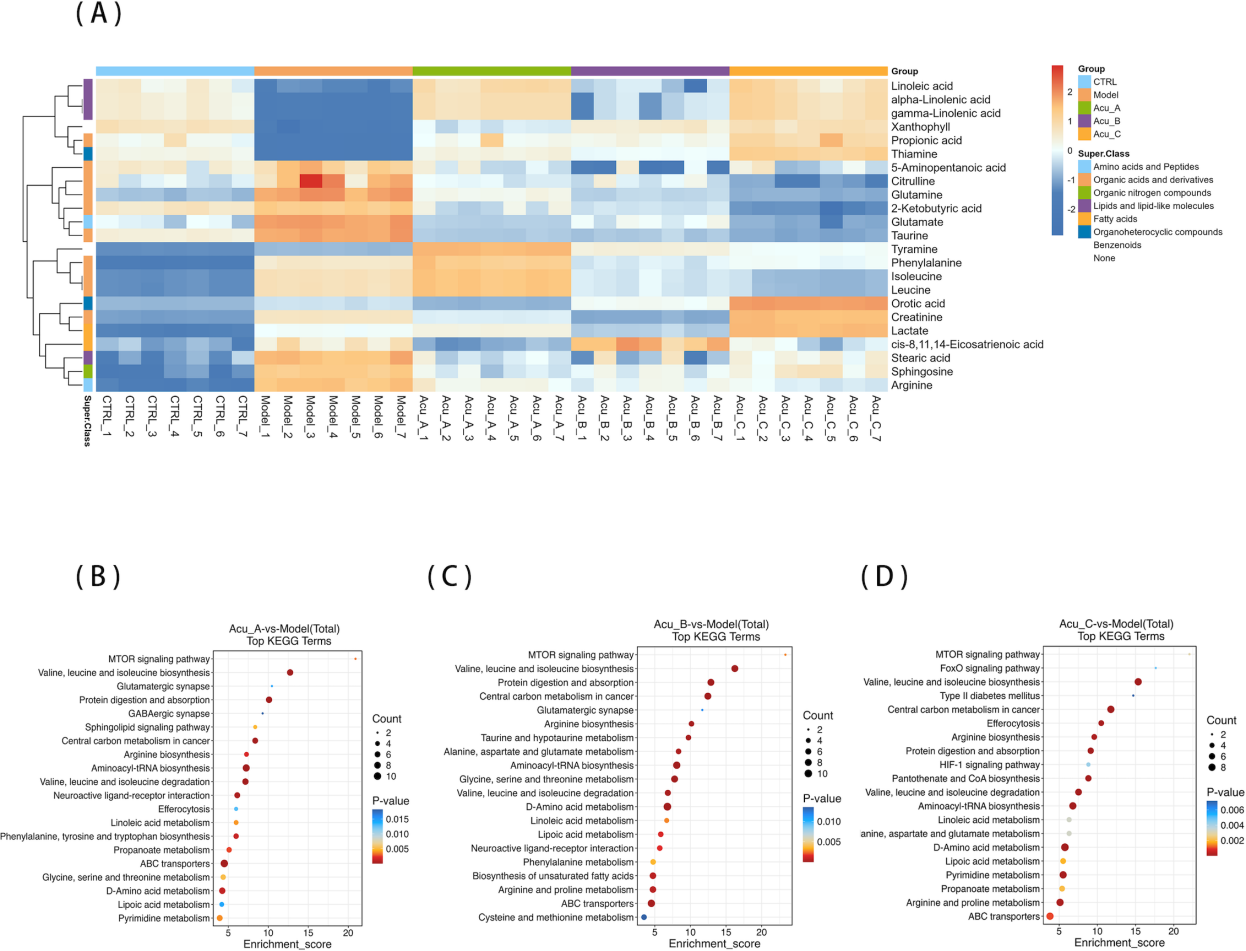
The effects of the three acupoint groups on the intestinal microbial community and host co-metabolism in IBD mice. **(A)** Heatmap of differential metabolites in the three acupoint groups. **(B-D)** Bubble plots of the top 20 differential metabolic pathways in the three acupoint groups. **Supplementary Figure 1**: Graphical abstract. Created with BioGDP.com.

The heatmap (**Figure 8A**) indicates pronounced metabolic abnormalities in the model group compared with controls. Beneficial metabolites such as propionic acid and linoleic acid were downregulated, whereas inflammation-associated metabolites (e.g., taurine, sphingosine, and glutamate) were increased. This abnormal pattern shifted toward normalization after intervention across all three acupoint groups. Propionic acid decreased in the model group, increased in all acupoint groups, whereas taurine, elevated in the model group, was markedly reduced. Moreover, each acupoint group exhibited a distinct metabolic signature. Specifically, Dachangshu primarily affected organic nitrogen compounds; Tianshu influenced organic acids and derivatives; and Shangjuxu were associated with changes in lipids and lipid-like molecules.

To further evaluate the metabolic effects of the three acupoints, KEGG pathway enrichment was performed on differentially expressed metabolites to summarize pathway-level regulation across groups. Among the top 20 enriched pathways, 11 were shared by all three acupoints (**Figures 8B–D**; **Table 1**). These shared pathways include Lipoic acid metabolism, D-Amino acid metabolism, ABC transporters, Linoleic acid metabolism, Valine, leucine and isoleucine degradation, Aminoacyl-tRNA biosynthesis, Arginine biosynthesis, Central carbon metabolism in cancer, Protein digestion and absorption, Valine, leucine and isoleucine biosynthesis, and the mTOR signaling pathway, spanning amino acid metabolism, lipids/fatty acids, and energy metabolism. For the Dachangshu point, enriched pathways included Phenylalanine, tyrosine and tryptophan biosynthesis, Sphingolipid signaling pathway, and GABAergic synapse. For the Tianshu point, enriched pathways included Cysteine and methionine metabolism, Biosynthesis of unsaturated fatty acids, Amino acid metabolism, and Taurine and hypotaurine metabolism. For the Shangjuxu point, enriched pathways included Pantothenate and CoA biosynthesis, HIF-1 signaling pathway, Type II diabetes mellitus, and FoxO signaling pathway.

**Table 1:** The top 20 differential metabolic pathways of the three acupoint groups.

Pathways	Group		
	AcuA&AcuB&AcuC		
Identical pathways	Lipoic acid metabolism D-Amino acid metabolism ABC transporters Linoleic acid metabolism Valine, leucine and isoleucine degradation Aminoacyl-tRNA biosynthesis Arginine biosynthesis Central carbon metabolism in cancer Protein digestion and absorption Valine, leucine and isoleucine biosynthesis mTOR signaling pathway		
	AcuA&AcuB	AcuB&AcuC	AcuA&AcuC
Identical pathways	Neuroactive ligand-receptor interaction	Arginine and proline metabolism	Propanoate metabolism
	Glycine, serine and threonine metabolism	Alanine, aspartate and glutamate metabolism	Pyrimidine metabolism
	Glutamatergic synapse		Efferocytosis
	AcuA	AcuB	AcuC
Unique pathways	Phenylalanine, tyrosine and tryptophan biosynthesis	Cysteine and methionine metabolism	Pantothenate and CoA biosynthesis
	Sphingolipid signaling pathway	Biosynthesis of unsaturated fatty acids	HIF-1 signaling pathway
	GABAergic synapse	Amino acid metabolism	Type II diabetes mellitus
		Taurine and hypotaurine metabolism	FoxO signaling pathway

## 4 Discussion

IBD arises from disruptions in host-microbiota interactions driven by microbial and environmental factors (Khor et al., 2011). In clinical studies of IBD, acupuncture has been shown to markedly reduce endoscopic severity indices, histological scores, and recurrence rates, and to increase the relative abundance of gut bacteria (Bao et al., 2022). Acupoint selection materially affects IBD management, yet despite the efficacy of electroacupuncture, the optimal acupoints and the mechanisms underlying differential efficacy remain to be defined.

In this experimental IBD mouse model, all three acupoints tended to mitigate weight loss and intestinal inflammation and also modulated circulating inflammatory markers. Immune cell-derived cytokines orchestrate the inflammatory response, and pro-inflammatory cytokines drive chronic intestinal inflammation, tissue damage, malignancy, and disease persistence while impeding recovery (Neurath, 2024). IL-1β is a key modulator of inflammation, reflecting host defense against infection while exacerbating chronic disease (Lopez-Castejon and Brough, 2011). IL-6 promotes T-cell survival and apoptosis resistance in the inflamed lamina propria, expanding CD4+ T cells and perpetuating inflammation (Mudter and Neurath, 2007). In this study, serum IL-6 and IL-1β levels were significantly higher in IBD mice than in controls. Following electroacupuncture at the three acupoints, IL-1β levels did not differ significantly from the model group. By contrast, IL-6 levels were significantly lower in all three acupoint groups than in the model group. Among them, the Dachangshu group exhibited the lowest IL-6 levels.

IBD development is closely linked to gut dysbiosis, characterized by reduced species diversity and enrichment of pathogenic strains that amplify inflammation and compromise intestinal barrier integrity (Ahlawat et al., 2021; Kumbhari et al., 2024). Regulatory modulation of the gut microbiota has been documented, with animal studies showing that electroacupuncture markedly reduces Lachnoclostridium, Lachnospiraceae_UCG-006, Odoribacter, and Oscillibacter (An et al., 2022). Electroacupuncture can also modulate the Firmicutes/Bacteroidetes (F/B) ratio in patients with hypertension (Wang et al., 2021). The F/B ratio is considered important for intestinal homeostasis, and deviations in either direction may indicate dysbiosis (Stojanov et al., 2020). In this experiment, the model group exhibited a higher F/B ratio than the control group. Following electroacupuncture, the F/B ratio in the Dachangshu group was lower than that in the model group.

Our study found that electroacupuncture at all three acupoints was associated with shifts in intestinal microbiota dysbiosis in IBD mice. Community composition histograms showed varying degrees of divergence from the model group across the three acupoint groups. Specifically, at the phylum level, the relative abundances of Proteobacteria and Verrucomicrobiota in the Shangjuxu group differed from the model group. At the genus level, Akkermansia and Bacteroides abundances in the Shangjuxu group differed significantly from the model group. Verrucomicrobiota is a major bacterial phylum; Akkermansia, its most studied genus, is considered a promising probiotic candidate. IBD patients exhibit markedly reduced mucosal Akkermansia compared with healthy individuals (Cani et al., 2022). Proteobacteria are widely recognized as indicators of gut dysbiosis, and abnormal increases are among the most reliable signs of microbial imbalance. Studies show increased Proteobacteria in IBD, including Escherichia coli, Campylobacter, and Helicobacter, which contribute to disease pathophysiology (Mukhopadhya et al., 2012). We therefore hypothesize that electroacupuncture may ameliorate IBD by reshaping gut microbiota structure, increasing beneficial taxa, and reducing potentially harmful taxa.

The gut microbiota role in IBD pathophysiology is well established, with microbial metabolites influencing immune maturation and homeostasis, host energy metabolism, and mucosal integrity; notably, the roles of short-chain fatty acids, tryptophan metabolites, and bile acids have been demonstrated experimentally (Lavelle and Sokol, 2020). In the gut, tryptophan is metabolized primarily via the serotonin, kynurenine, and indole derivative pathways. These pathways are regulated by gut bacteria directly or indirectly (Agus et al., 2018). In this study, electroacupuncture at all three acupoints converged on shared pathways, including Lipoic acid metabolism, D-Amino acid metabolism, ABC transporters, Linoleic acid metabolism, Valine, leucine and isoleucine degradation, Aminoacyl-tRNA biosynthesis, Arginine biosynthesis, Central carbon metabolism in cancer, Protein digestion and absorption, Valine, leucine and isoleucine biosynthesis, and the mTOR signaling pathway. These pathways encompass amino acids and their metabolites, lipids and fatty acids, and energy metabolism. These findings suggest that electroacupuncture-associated metabolic changes may engage these networks and relate to alterations in microbial-host co-metabolism implicated in IBD pathogenesis.

Amino acids are essential for maintaining immune function and disease resistance, with anti-inflammatory, antioxidative, and anti-apoptotic properties (Liu et al., 2017). Amino acids are absorbed primarily in the distal small intestine, whereas the large intestine takes up amino acids derived from bacterial metabolism and endogenous sources (Bröer, 2023). In IBD, mucosal injury disrupts barrier integrity and downregulates amino acid transporters, and diminished tryptophan transporter activity further exacerbates intestinal inflammation. Tryptophan also acts as an upstream regulator in the resolution of intestinal inflammation (Zheng et al., 2023). Tryptophan metabolites can bind and activate the aryl hydrocarbon receptor, inducing downstream cytokines such as IL-22 and IL-17, thereby helping maintain intestinal homeostasis in IBD (Sun et al., 2020). Our data indicate that electroacupuncture at all three acupoints in the IBD model mice is accompanied by alterations in amino acid metabolism. These alterations may involve shifts in key pathways, including valine, leucine and isoleucine degradation and arginine biosynthesis.

Across all three electroacupuncture groups, significant alterations were observed in the linoleic acid metabolism pathway, accompanied by changes in lipid and fatty acid metabolism. 10-Hydroxy-cis-12-octadecenoic acid (HYA), a microbial metabolite of linoleic acid, modulates tight junction-associated molecules under DSS challenge, downregulates TNFR2 in mouse intestinal epithelial cells, upregulates G protein-coupled receptor 40 (GPR40) in Caco-2 cells, and may confer therapeutic benefits in IBD and related disorders (Miyamoto et al., 2015). Arachidonic acid, a key product of linoleic acid metabolism and an essential precursor of inflammatory mediators, shows elevated levels in the intestinal mucosa of patients with IBD (Huss et al., 2025). Arachidonic acid, a polyunsaturated fatty acid, is converted to prostaglandins via cyclooxygenase (COX) and downstream synthases. COX exists as two isoforms, COX-1 and COX-2, which have distinct roles in health and disease. COX-1 is constitutively expressed across tissues and supports physiological homeostasis. COX-2 is induced during inflammation, driving prostaglandin production that mediates pain and promotes inflammatory processes, and has been linked to IBD symptoms such as abdominal pain and diarrhea (Simon, 1999).

Analyses indicated possible alterations in three metabolic pathways in the Dachangshu acupoint group: phenylalanine, tyrosine and tryptophan biosynthesis, sphingolipid signaling, and GABAergic synapse. These pathways are closely linked to neuro-immune modulation. Notably, the Dachangshu point was associated with changes in the sphingolipid signaling pathway. Sphingolipids are membrane structural components and key signaling molecules in eukaryotic cells. As regulators of inflammation and immunity, sphingolipids have been identified among the most differentially expressed fecal metabolites in IBD (Brown et al., 2019). Ceramides are core sphingolipids involved in apoptosis and differentiation, whereas sphingosine-1-phosphate (S1P) is a key signaling lipid in proliferation and inflammation, all essential for cellular function. IBD is associated with dysregulated sphingolipid metabolism, and S1P plays a critical role in inflammatory signaling and pro-survival pathways (Espinoza and Snider, 2024).

The brain and gut function as interconnected sensory organs, perceiving, transmitting, and integrating signals from both internal and external environments. Research highlights the gut-brain axis as a critical modulator of inflammatory nociception, inflammatory responses, and immune homeostasis (Agirman et al., 2021). Neuroimaging studies reveal that patients with IBD often present with structural and functional CNS changes, potentially explaining the high prevalence of depressive symptoms in this population (Wang et al., 2023). The co-occurrence of IBD and depression may be associated with dysregulation of GABAergic synaptic pathways. Studies on electroacupuncture for IBD indicate it modulates hippocampal inflammation and metabolism, reducing gut microbiota impact and alleviating anxiety- and depression-like behaviors in IBD model rats (Zhou et al., 2022). Additionally, abdominal pain in IBD is linked to gut-brain axis interaction. Gamma-aminobutyric acid (GABA) is viewed as a potential therapeutic candidate for IBD symptoms, given its regulation of visceral pain and beneficial effects on the digestive tract (Lucarini et al., 2024). In the Dachangshu acupoint group, exploratory analysis suggested changes in GABAergic synapses. This finding offers preliminary insights into the potential involvement of gut-brain axis mechanisms in IBD.

In the Tianshu acupoint group, changes were noted in four pathways: cysteine and methionine metabolism, biosynthesis of unsaturated fatty acids, amino acid metabolism, and taurine and hypotaurine metabolism. Amino acids are crucial for maintaining physiological functions and metabolic homeostasis. Persistently high levels of branched-chain amino acids (BCAAs) can precipitate metabolic disorders. Their downstream metabolites can trigger inflammatory responses and lipotoxicity in metabolic tissues, resulting in various metabolic dysregulations (Mansoori et al., 2025). Fatty acids serve multiple roles in IBD, including anti-inflammatory and immunomodulatory effects, modulation of gut microbiota, and maintenance of the intestinal barrier. Short-chain fatty acids (SCFAs) are known for their potent anti-inflammatory effects and may play a vital role in preventing IBD. In contrast, long-chain fatty acids, including saturated, trans, and omega-6 polyunsaturated varieties, are associated with pro-inflammatory effects. Medium- and very-long-chain fatty acids have the ability to regulate inflammation, mucosal barriers, and gut microbiota (Yan et al., 2023). In the Tianshu acupoint group, significant changes were observed in the above-mentioned amino acid and lipid metabolism, and these changes were associated with the key pathways related to energy balance.

In the Shangjuxu acupoint group, changes were noted in four pathways: pantothenate and CoA biosynthesis, HIF-1 signaling, Type II diabetes mellitus, and FoxO signaling. Notably, the Shangjuxu acupoint influences both HIF-1 and FoxO signaling pathways. FoxO regulates apoptosis, autophagy, oxidative stress, and the release of inflammatory cytokines, contributing to normal cellular physiology (Kim et al., 2022). Dynamic variations in oxygen tension within intestinal cells are crucial for maintaining oxygen homeostasis, with HIF playing a vital role in intestinal hypoxia. Hypoxia may arise under regular physiological conditions or from functional impairments, such as inflammatory hypoxia. Research indicates that HIF-1 may hold therapeutic potential for targeting hypoxia-related signaling pathways in intestinal disorders (Dvornikova et al., 2023). Changes were observed in all the aforementioned pathways in the Shangjuxu acupoint group. This finding suggests that these changes may be related to alterations in the cellular environment and the overall metabolic state, and are further associated with the improvement of IBD.

Our experimental results indicate that electroacupuncture treatment at these three acupoints is associated with improvements in inflammatory markers in IBD mice. The metabolic pathways shared by these three acupoints include amino acids and their metabolites, lipids and fatty acids, and energy metabolism. At the same time, distinct metabolic profiles were observed for each group of acupoints. Specifically, changes in the Dachangshu point group involve pathways related to brain signal transmission; changes in the Tianshu point group involve pathways related to amino acid and lipid metabolism; and changes in the Shangjuxu point group involve pathways related to the cellular environment and immune status.

We postulate that the differences in acupoint locations may correlate with the biological processes they influence. The Dachangshu acupoint, located on the back near spinal nerve segments, may be linked to the observed changes in neural and inflammatory pathways. The Tianshu acupoint, situated in the abdomen near the intestines—the primary organs affected by IBD—may be more closely related to the alterations in gastrointestinal metabolic pathways noted in this condition. The Shangjuxu acupoint, located on the lower leg, exerts a systemic modulatory effect on IBD, potentially linked to neurohormonal regulation and the maintenance of systemic homeostasis.

## 5 Conclusion

Electroacupuncture is a commonly used external treatment method, which has shown efficacy related to acupoints in mouse models of IBD. Stimulation of BL25, ST25, and ST37 did not significantly alter IL-1β, while BL25 reduced IL-6, indicating differences in the regulation of inflammatory cytokines. These three acupoints all altered the intestinal microbiota, amino acids, lipids, and energy metabolism, with differences in the metabolic pathways of Dachangshu, Tianshu, and Shangjuxu acupoints. Since these findings are correlational and all animals were anesthetized with isoflurane, they provide preliminary insights into the selection of acupoints. Future studies should conducted with sham controls, non−anesthetized conditions, and causal approaches (e.g., fecal microbiota transplantation, metabolite supplementation, pathway inhibition) to further elucidate the underlying mechanisms.

## Data Availability

The datasets presented in this study can be found in online repositories. The names of the repository/repositories and accession number(s) can be found in the article/**Supplementary Material**.

## Supplementary material

Supplementary Table 1 Validation parameters of OPLS-DA models for each comparison **Supplementary Table 1**: Models were validated by 7-fold cross-validation (Q²) and permutation tests (n=200). R²Y(cum), explained variation in group membership; Q²(cum), predicted variation; Permutation (Q² intercept), intercept of the permuted Q² regression line.
