# Supplementary figures and images for: Evaluation of electroacupuncture acupoint selection’s potential in the treatment of inflammatory bowel disease: an investigation using a mouse gut microbiome and metabolomics model
Source: Front Cell Infect Microbiol. 2026 Jun 10;16:1830455. doi: 10.3389/fcimb.2026.1830455 (PMC13291076; doi:10.3389/fcimb.2026.1830455)

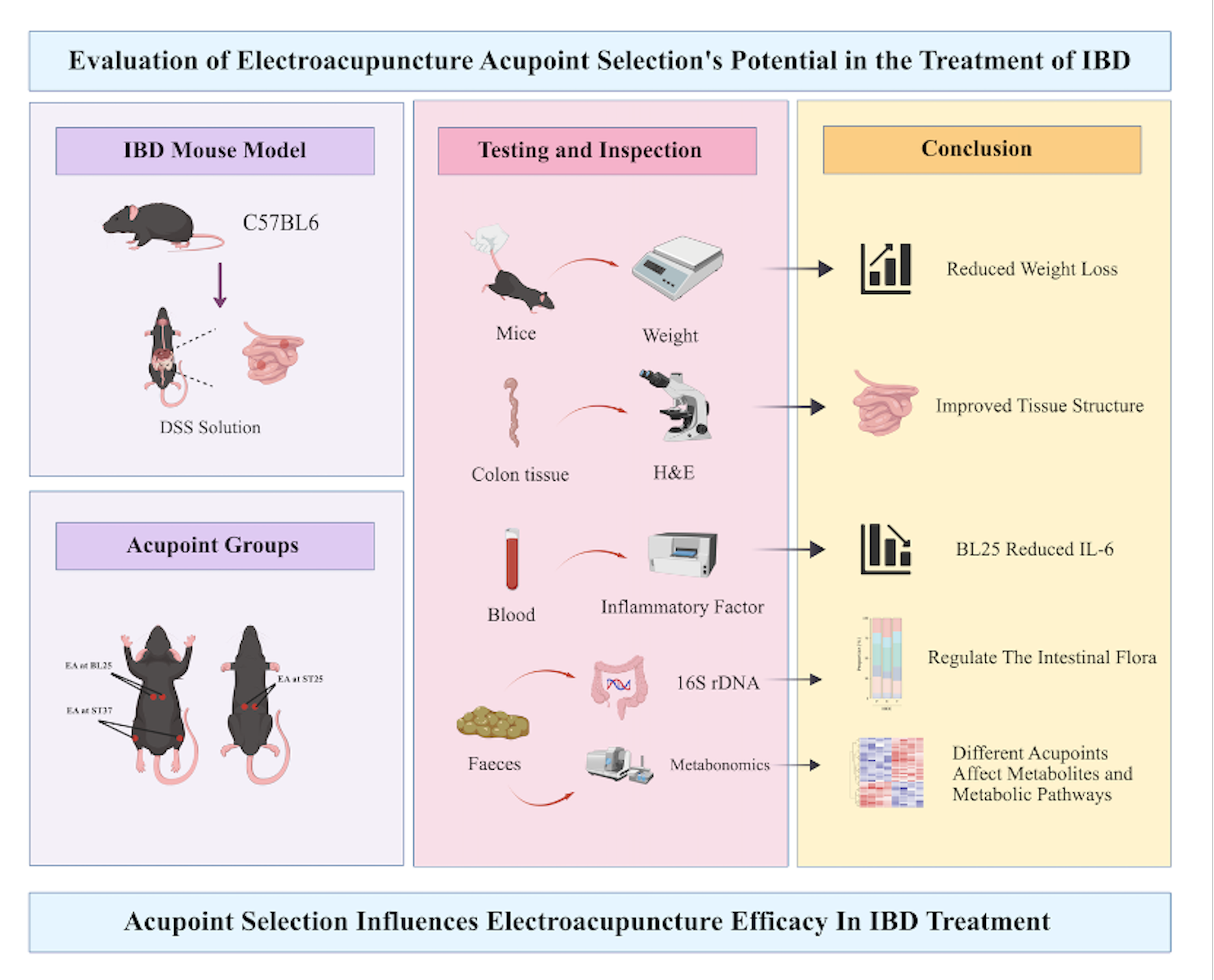

Supplement: Supplementary file 4 [file Image1.tiff]
